# BMS-470539 Attenuates Oxidative Stress and Neuronal Apoptosis via MC1R/cAMP/PKA/Nurr1 Signaling Pathway in a Neonatal Hypoxic-Ischemic Rat Model

**DOI:** 10.1155/2022/4054938

**Published:** 2022-01-31

**Authors:** Shufeng Yu, Desislava Met Doycheva, Marcin Gamdzyk, Yuanyuan Gao, Yong Guo, Zachary D. Travis, Jiping Tang, Wen-Xiong Chen, John H. Zhang

**Affiliations:** ^1^Department of Neurology, Guangzhou Women and Children's Medical Center, Guangzhou Medical University, Guangzhou 510623, China; ^2^Department of Physiology and Pharmacology, Basic Sciences, School of Medicine, Loma Linda University, Loma Linda, CA 92354, USA; ^3^Cerebrovascular Center, Henan Provincial People's Hospital, Zhengzhou University, Zhengzhou 450003, China; ^4^Department of Anesthesiology, Neurosurgery and Neurology, Loma Linda University School of Medicine, Loma Linda, CA 92354, USA

## Abstract

Neuronal apoptosis induced by oxidative stress plays an important role in the pathogenesis and progression of hypoxic-ischemic encephalopathy (HIE). Previous studies reported that activation of melanocortin-1 receptor (MC1R) exerts antioxidative stress, antiapoptotic, and neuroprotective effects in various neurological diseases. However, whether MC1R activation can attenuate oxidative stress and neuronal apoptosis after hypoxic-ischemic- (HI-) induced brain injury remains unknown. Herein, we have investigated the role of MC1R activation with BMS-470539 in attenuating oxidative stress and neuronal apoptosis induced by HI and the underlying mechanisms. 159 ten-day-old unsexed Sprague-Dawley rat pups were used. HI was induced by right common carotid artery ligation followed by 2.5 h of hypoxia. The novel-selective MC1R agonist BMS-470539 was administered intranasally at 1 h after HI induction. MC1R CRISPR KO plasmid and Nurr1 CRISPR KO plasmid were administered intracerebroventricularly at 48 h before HI induction. Percent brain infarct area, short-term neurobehavioral tests, Western blot, immunofluorescence staining, Fluoro-Jade C staining, and MitoSox Staining were performed. We found that the expression of MC1R and Nurr1 increased, peaking at 48 h post-HI. MC1R and Nurr1 were expressed on neurons at 48 h post-HI. BMS-470539 administration significantly attenuated short-term neurological deficits and infarct area, accompanied by a reduction in cleaved caspase-3-positive neurons at 48 h post-HI. Moreover, BMS-470539 administration significantly upregulated the expression of MC1R, cAMP, p-PKA, Nurr1, HO-1, and Bcl-2. However, it downregulated the expression of 4-HNE and Bax, as well as reduced FJC-positive cells, MitoSox-positive cells, and 8-OHdG-positive cells at 48 h post-HI. MC1R CRISPR and Nurr1 CRISPR abolished the antioxidative stress, antiapoptotic, and neuroprotective effects of BMS-470539. In conclusion, our findings demonstrated that BMS-470539 administration attenuated oxidative stress and neuronal apoptosis and improved neurological deficits in a neonatal HI rat model, partially via the MC1R/cAMP/PKA/Nurr1 signaling pathway. Early administration of BMS-470539 may be a novel therapeutic strategy for infants with HIE.

## 1. Introduction

Neonatal hypoxic-ischemic encephalopathy (HIE) is hypoxic-ischemic (HI) brain injury caused by perinatal asphyxia in neonates, which is one of the leading causes of morbidity and mortality in infants [1–3]. HIE affects 1–8 cases per 1000 live births and accounts for 23% of all neonatal deaths globally [1, 4]. Those who survive suffer permanent neurodevelopmental deficits such as cerebral palsy, developmental delay, intellectual disability, cognitive deficits, and epilepsy [5, 6]. Thus far, there is still a lack of effective treatments for HIE [7, 8], which makes exploring new therapeutic targets necessary.

Strong evidence indicates that oxidative stress and neuronal apoptosis play a crucial role in the pathogenesis and progression of hypoxic-ischemic-induced brain injury [9, 10]. The newborn brain is susceptible to hypoxia and requires high levels of oxygen supply [11]. Following hypoxia and ischemia, the production of reactive oxygen species (ROS) increases rapidly and exceeds the capacity of the antioxidant clearance system, resulting in oxidative stress [12, 13]. Moreover, the generation of oxidative stress triggers the release of free radicals, apoptosis, and necrosis, which eventually leads to secondary neuronal damage after HIE [11, 14, 15]. Oxidative stress-mediated apoptosis has been reported to be an important neuronal death mechanism of HIE [11, 16]. Over the past decade, mounting evidence has demonstrated that inhibiting oxidative stress and neuronal apoptosis have a neuroprotective effect in neonatal HIE animal models [9, 10, 17].

The melanocortin-1 receptor (MC1R) has been identified as a G-protein coupled receptor (GPCR) expressed in melanoma cells and melanocytes, which plays an important role in melanoma and skin pigmentation by inhibiting ultraviolet radiation- (UVR-) induced apoptosis, oxidative stress, and DNA damage [18, 19]. The expression of MC1R was also reported in the central nervous system (CNS), including neurons, astrocytes, and microglia [20]. Recent studies suggested that the activation of MC1R suppressed mitochondria-mediated oxidative stress and neuronal apoptosis after intracerebral hemorrhage (ICH) in mice [21]. MC1R activation also exerted anti-inflammatory, antiapoptotic, and neuroprotective effects in a mice model of experimental traumatic brain injury [22]. As a synthesized small-molecule and specific selective agonist of MC1R, 1-[1-(3-methyl-L-histidyl-O-methyl-D-tyrosyl)-4-phenyl-4-piperidinyl]-1-butanone (BMS-470539) has been shown to attenuate early brain injury (EBI) following subarachnoid hemorrhage (SAH) by inhibiting oxidative stress, neuronal apoptosis, and mitochondrial fission via MC1R signaling [23, 24]. By binding with MC1R, BMS-470539 suppressed lipopolysaccharide- (LPS-) induced inflammatory responses and delayed neutrophil apoptosis [25]. In our previous study, we have shown that activation of the MC1R with BMS-470539 attenuated neuroinflammation after neonatal hypoxic-ischemic brain injury in rats [26]. Specifically, BMS-470539 administration significantly increased intracellular cAMP and Nurr1 expression levels [26].

Nurr1, also known as NR4A2, is expressed in CNS and other peripheral tissues [26, 27]. Nurr1 has been reported to protect neurons from oxidative stress-induced death by inhibiting apoptosis in vitro and in vivo Parkinson's disease (PD) models [28, 29]. Furthermore, mounting evidence has demonstrated that downregulation of Nurr1 with small interfering RNA- (siRNA-) mediated gene silencing promotes apoptosis by increasing the expression of proapoptotic proteins, such as Bax [30, 31]. Nurr1 is strongly induced via the cAMP/PKA-dependent pathway [32, 33]. The cAMP/PKA signaling pathway plays critical roles in mediating physiological processes and pathophysiological mechanisms, including growth, differentiation, metabolism, inflammation, oxidative stress, and apoptosis in various types of cells [26, 34–36]. Moreover, MC1R attenuates ultraviolet-induced oxidative stress and promotes DNA repair after ultraviolet by cAMP-dependent signaling pathway [19, 37]. In our previous study, it has been indicated that Nurr1 is the downstream protein contributing to the MC1R mediated anti-inflammatory effect [26]. The role and molecular mechanisms of MC1R and Nurr1 on oxidative stress and apoptosis after neonatal HIE have never been investigated.

Therefore, the present study is aimed at exploring whether the selective MC1R agonist, BMS-470539, could attenuate oxidative stress and neuronal apoptosis via MC1R/cAMP/PKA/Nurr1 signaling pathway after neonatal hypoxic-ischemic brain injury in rats.

## 2. Materials and Methods

### 2.1. Animals

In this study, post-natal day 5-6 unsexed Sprague-Dawley (SD) rat pups were purchased from Harlan Labs (Livermore, CA) with their mothers who were housed in a humidity- and temperature-controlled environment until they reached post-natal day 10 (P10), with a regular 12 h light and dark cycle. A total of 159 rat pups regardless of gender, weighing 14~20 g, were used in this study. All P10 neonatal pups were randomly assigned to either the sham group (*n* = 36) or HI surgery group (*n* = 123). However, 3 of 120 HI surgery rat pups were excluded from the study due to death during or after hypoxia. The Institutional Animal Care and Use Committee at Loma University approved all the experimental procedures and protocols for this study, which comply with the National Institutes of Health (NIH) Guide for the Care and Use of Laboratory Animals and ARRIVE guidelines (Animal Research: Reporting of In Vivo Experiments).

### 2.2. Experimental Design

#### 2.2.1. Experiment 1

The time course expression of endogenous MC1R and Nurr1 were evaluated at sham, 6 h, 12 h, 24 h, 48 h, 72 h, and 7 d post-HI. The rat pups were randomly divided into 7 groups (*n* = 6/group): sham, 6 h HI, 12 h HI, 24 h HI, 48 h HI, 72 h HI, and 7 d HI. Brain samples of the right (ipsilateral) hemisphere were collected for Western blot analysis. The sham group rat pups were euthanized at 48 h post-HI.

#### 2.2.2. Experiment 2

The effects of the exogenous MC1R agonist BMS-470539 was evaluated at 48 h post-HI. The optimal dose of BMS-470539 (160 *μ*g/kg, sc-362716A, Santa Cruz, USA) was selected based on our previous study [26], which was used for the following short-term outcome and mechanism studies. The rat pups were randomly divided into 3 groups (*n* = 6/group): sham, HI+vehicle, and HI+BMS-470539. BMS-470539 (160 *μ*g/kg) or vehicle (sterile saline) were administered intranasally at 1 h post-HI. Infarct area, short-term neurobehavioral tests: negative geotaxis, and body weight were evaluated at 48 h post-HI.

#### 2.2.3. Experiment 3

The colocalization of MC1R and Nurr1 on neurons was evaluated using double immunofluorescence at 48 h post-HI. Moreover, to evaluate the effects of MC1R activation with BMS-470539 treatment on neuronal apoptosis, double immunofluorescence staining of C-Cas 3 with neuronal nuclei (NeuN, a marker for neurons) was conducted at 48 h post-HI. The rat pups were randomly divided into 3 groups (*n* = 4/group): sham, HI+vehicle (sterile saline), and HI+BMS-470539 (160 *μ*g/kg).

#### 2.2.4. Experiment 4

To study the neuroprotective mechanism of MC1R activation, the rat pups were randomly assigned to 6 groups (*n* = 6/group): sham, HI+vehicle, HI+BMS-470539, HI+BMS-470539+MC1R KO CRISPR, HI+BMS-470539+Nurr1 KO CRISPR, and HI+BMS-470539+control CRISPR. BMS-470539 (160 *μ*g/kg) or vehicle (sterile saline) were injected intranasally 1 h post-HI. MC1R CRISPR, Nurr1 CRISPR, and control CRISPR were given via intracerebroventricular injection at 48 h before HI. Fluoro-Jade C staining (FJC), 8-hydroxy-2′-deoxyguanosine (8-OHdG) staining, MitoSox staining, and Western blot were conducted at 48 h post-HI. Moreover, to evaluate the knockout efficiency of MC1R KO CRISPR and Nurr1 KO CRISPR, an additional 24 rat pups were randomly divided into 6 groups (*n* = 4/group): naive+control CRISPR, naive+MC1R KO CRISPR, naive+Nurr1 KO CRISPR, HI+control CRISPR, HI+MC1R KO CRISPR, and HI+Nurr1 KO CRISPR. The right (ipsilateral) hemisphere of each brain was collected for Western blot analysis at 48 h post-HI.

### 2.3. Neonatal HIE Model

The neonatal HI model was performed using the well-established Rice Vannucci model as previously described [38]. Briefly, P10 rat pups were anesthetized with 3% isoflurane and maintained throughout the surgery with 2.5%. After aseptic preparation, a longitudinal midline neck incision was made in the right anterior neck. The right common carotid artery was identified and isolated gently from the surrounding structures. It was then double ligated with a 5.0 silk surgical suture and severed between the ligation sites. The right common carotid artery was exposed for the sham group rat pups but without ligation and hypoxic conditions. The total time taken per surgery was 5-9 minutes, after which the pups were allowed to recover for 1 h on temperature-controlled heating blankets. Pups were placed in an airtight jar partially submerged in a 37°C water bath. Following this, rat pups were exposed to a gas mixture of 8% oxygen and 92% nitrogen for 2 h and 30 min. After hypoxic treatment, the pups were returned to their mothers.

### 2.4. Drug Administration

#### 2.4.1. Intranasal Drug Administration

As a promising route of administration for CNS targeting, intranasal drug administration has several advantages, including bypassing the blood-brain barrier, targeting the brain directly, shorter time to onset of effect, and higher bioavailability due to avoidance of hepatic first-pass metabolism [39, 40]. Previous studies and our data showed that BMS-470539 administration 1 h after SAH or HI via the intranasal pathway exerts neuroprotective effects in the SAH and HIE rat models [20, 24, 26]. Based on the above studies, BMS-470539 was also administered intranasally at 1 h after HI induction in the present study. Briefly, the anesthetized rat pups were placed on their backs and given either BMS-470539 (160 *μ*g/kg, sc-362716A, Santa Cruz) or vehicle (sterile saline) intranasally at 1 h post-HI within 10 min. 2 *μ*l per drop was administered every 2 min, alternating between the left and right nares. A total volume of 10 *μ*l was administered intranasally.

#### 2.4.2. Intracerebroventricular Drug Administration

For the mechanism experiment, the engineered form of the CRISPR-associated (Cas9) protein system was performed in the study. Briefly, the CRISPR protein Cas9 is directed to genomic target sites by specific short guide RNAs, where it functions as an endonuclease, and further inactivates or activates specific gene [41]. MC1R CRISPR KO plasmid (Santa Cruz Biotechnology, USA), Nurr1 CRISPR KO plasmid (Santa Cruz Biotechnology, USA), or control CRISPR plasmid (Santa Cruz Biotechnology, USA) was given via intracerebroventricular injection at 48 h before HI induction according to our previous study [26]. Western blot was performed to evaluate the knockout efficiency of knockout CRISPR. As previously described [17, 42], rat pups were anesthetized with isoflurane (3% induction, 2.5% maintenance) and then placed in a stereotaxic frame. A burr hole was drilled into the skull, and a 10 *μ*l Hamilton syringe (Hamilton Company, USA) needle was inserted through the burr hole into the left lateral ventricle at 1.5 mm posterior, 1.5 mm lateral to the bregma, and 1.7 mm deep into the ipsilateral hemisphere. A total of 2 *μ*l per rat pup of CRISPR was slowly administered intracerebroventricularly (i.c.v) at a rate of 0.3 *μ*l/min using an infusion pump. Thereafter, the needle was kept in situ for an additional 10 min to prevent leakage and backflow and then retracted slowly over 5 min. The burr hole was sealed with bone wax, and the skin was closed with sutures.

### 2.5. Neurological Evaluation

To assess short-term neurological function, as previously described, negative geotaxis test was conducted by two blinded investigators in an unbiased setup at 48 h post-HI [43]. Negative geotaxis was performed by placing the rat pups head downward on an inclined board (45°), and the time it took for the rat pups to turn their bodies to head upward position was recorded. The maximum testing time was 60 s [17].

### 2.6. Infarct Area Measurements

The rat pups were anesthetized and then euthanized at 48 h post-HI. 2,3,5-Triphenyltetrazolium chloride monohydrate (TTC) (Sigma Aldrich, USA) staining, a standard and reliable method to evaluate the infarct area in ischemic stroke models [17, 44, 45], was used to evaluate percent infarction. The brains were removed and sectioned into 2 mm coronal brain slices, and a total of 5 brain slices were prepared as previously described [17, 43]. These coronal brain slices were incubated in 2% TTC solution for 5 min at room temperature. After being washed with PBS and fixed in 10% formaldehyde solution overnight, these brain slices were then imaged to measure and analyze the infarct area using ImageJ software (NIH, USA). The percentage of infarct area for each brain slice was calculated as follows: [(total area of contralateral hemisphere) − (area of non − infarcted area of ipsilateral hemisphere)]/(total area of contralateral hemisphere × 2)∗100% [17, 43, 46].

### 2.7. Western Blotting Analysis

Western blot was performed as previously described [17, 43, 47]. The rat pups were euthanized with isoflurane and then perfused with 4°C phosphate-buffered saline (PBS) at 48 h post-HI. The brains were removed and instantly divided into the ipsilateral and contralateral cerebrums, snap frozen in liquid nitrogen, and then stored in a -80°C freezer for Western blot. The brain samples of ipsilateral hemisphere was used to measure the protein expression in the neonatal HIE model [8, 17, 43, 46, 48]. To prepare samples for Western blot, the ipsilateral hemisphere tissue was homogenized in RIPA lysis buffer (Santa Cruz Biotechnology, USA) and further centrifuged at 14,000 g at 4°C for 20 min. A supernatant was collected, and protein concentration was measured using a detergent compatibility assay (Bio-Rad, Dc protein assay). After calculation of protein concentration of each sample using spectrophotometer (ThermoFisher Scientific, USA), equal amounts of protein (40 *μ*g) of protein were loaded into a 8%-12% SDS-PAGE gel for electrophoresis and then transferred onto a nitrocellulose membranes (0.45 *μ*m). The membranes were blocked with 5% nonfat blocking grade milk (Bio-Rad, USA) for 1 h at room temperature and then incubated overnight at 4°C with the following primary antibodies: MC1R (1 : 500, PA5-75342, ThermoFisher, USA), anti-Nurr1 (1 : 500, ab41917, Abcam, USA), anti-cAMP (1 : 1000, ab76238, Abcam, USA), p-PKA (1 : 1000, #4781S,Cell Signaling, USA), PKA (1 : 1000, #4782S, Cell Signaling, USA), anti-4-HNE (1 : 500, ab46545, Abcam, USA), anti-HO-1 (1 : 1000, ab68477, Abcam, USA), anti-Bax (1 : 500, sc-7480, Santa Cruz, USA), anti-Bcl-2 (1 : 1000, ab194583, Abcam, USA), and anti-*β*-actin (1: 3000, sc-47778, Santa Cruz, USA). The membranes were incubated at room temperature for 2 h with the appropriate secondary antibodies (1 : 2000, sc-2357, Santa Cruz, USA). Bands were visualized with ECL Plus chemiluminescence reagent kit (Amersham Bioscience, Arlington Heights, IL) and then quantified with the ImageJ software (NIH, USA). The results were displayed as relative density (grayscale value of the target proteins/*β*-actin or total proteins).

### 2.8. Histology and Immunohistochemistry

Rat pups were anesthetized (3% isoflurane) at 48 h post-HI and transcardially perfused with ice-cold PBS and 10% formalin. The brains were collected and postfixed with 10% formalin at 4°C for 24 h. Thereafter, the brains were dehydrated in a 20% sucrose solution at 4°C until they sank and then transferred into 30% sucrose solution at 4°C until they sank. The brain samples were frozen at -80°C after being embedded into OCT compound (Scigen Scientific, USA); then, 10 *μ*m thick coronal sections were cut at -20°C using a cryostat (CM3050S-3, Leica Microsystems, USA) and then mounted on glass slides for immunofluorescence staining, Fluoro-Jade C (FJC) staining, and MitoSox staining.

#### 2.8.1. Immunofluorescence Staining

Immunofluorescence staining was performed as described previously [43, 46, 49]. Slices were washed with PBS three times for 5-10 min, and then incubated with 0.3% Triton X-100 for 15 min at room temperature. Thereafter, the slices were rinsed with PBS three times for 5 min each. After being blocked with 5% donkey serum in PBS for 1 h at room temperature, these brain sections were incubated at 4°C overnight with primary antibody including: MC1R (1 : 50, PA5-75342, ThermoFisher, USA), anti-Nurr1 (1 : 50, ab41917, Abcam, USA), anti-Cleaved Caspase-3 (C-Cas 3; 1 : 50, 9661S, Cell Signaling, USA), 8-hydroxy-2′-deoxyguanosine antibody (8-OHdG; 1 : 200, ab62623, Abcam, USA), and anti-NeuN (1 : 100, ab104224, Abcam, USA). Then, the slices were washed with PBS and incubated with the appropriate secondary antibodies (1 : 200, Jackson ImmunoResearch, USA) for 2 h at room temperature. Thereafter, these sections were rinsed with PBS three times for 10 min and then covered with DAPI (Vector Laboratories Inc., USA). The slices were visualized and photographed using a fluorescence microscope Leica DMi8 (Leica Microsystems, Germany). To evaluate neuronal apoptosis, the number of C-Cas 3-positive neurons was identified and counted in three fields which from the different sections per rat brain. The positive cells were quantified under a microscopic field of 200x magnification and expressed as positive cells per square millimeter (cells/mm^2^). To assess the extent of oxidative stress DNA damage, the percentage of 8-OHdG-positive cells were quantified and averaged in three randomly selected fields per rat brain over a microscopic field of 200x magnification.

#### 2.8.2. Fluoro-Jade C Staining

To assess the degenerating neurons, Fluoro-Jade C (FJC) staining was performed using FJC Ready-to-Dilute Staining Kit (Biosensis, USA) according to previously reported [17, 48, 50]. Briefly, the slides were immersed in 1% sodium hydroxide solution for 5 min, then rinsed in 70% ethanol, and then distilled water for 2 min each. The slides were then incubated with 0.06% potassium permanganate solution for 10 min, followed by rinsing for 2 min in distilled water, and then transferred into a 0.0001% solution of FJC (Millipore, USA) for 10 min. The slides were washed three times with distilled water for 1 min each, dried in an incubator at 50°C for 5 min, then cleared in xylene for 5 min and covered with DPX (Sigma-Aldrich, USA). The sections were imaged and analyzed using a fluorescence microscope Leica DMi8 (Leica Microsystems, Germany) equipped with Leica Application Suite software. The number of FJC-positive neurons were counted under a microscopic field of 200x magnification; one field from each of the three different sections per brain were averaged and expressed as positive cells per square millimeter (cells/mm^2^).

#### 2.8.3. MitoSox Staining

To detect the mitochondrial superoxide level, MitoSox staining was conducted according to a previous report [9]. The brain slices were incubated with 5 *μ*mol/l MitoSox (ThermoFisher, USA) in the dark for 10 min and then washed with PBS three times for 5-10 min. Next, slices were covered with DAPI (Vector Laboratories Inc., USA). The slices were visualized and photographed using a fluorescence microscope Leica DMi8 (Leica Microsystems, Germany) and then analyzed using Leica Application Suite software. The percentage of MitoSox-positive cells were counted under a microscopic field of 200x magnification; each of the three sections per brain were averaged and then measured with ImageJ software (NIH, USA).

### 2.9. Statistical Analysis

All data were presented as the mean and standard deviation (mean ± SD). Statistical analysis was performed using GraphPad Prism 7(Graph Pad Software, San Diego, USA). The differences among multiple groups were analyzed using one-way ANOVA followed by Tukey's post hoc test. A *p* value < 0.05 was considered statistically significant.

## 3. Results

### 3.1. Time Course Expression Levels of Endogenous MC1R and Nurr1 Post-HI

**Figure 1:**
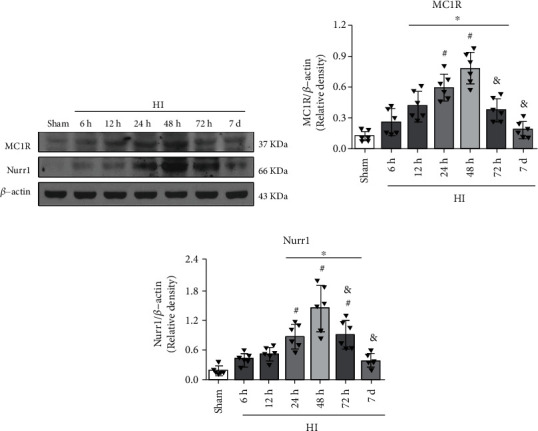
Temporal expression of endogenous MC1R and Nurr1 in the ipsilateral brain hemisphere post-HI. (a) Representative Western blot bands of the time course. (b) Western blot data showed that the endogenous expression levels of MC1R significantly increased from 12 h reaching peak at 48 h post-HI. (c) Nurr1 expression levels significantly increased over time, reached highest at the 48 h post-HI. *n* = 6 per group. Data were represented as mean ± SD. ^∗^*p* < 0.05 versus sham, ^#^*p* < 0.05 versus 6 h HI, and ^&^*p* < 0.05 versus 48 h HI; one-way ANOVA, Tukey's post hoc test.

Western blot was performed to evaluate the protein expression levels of MC1R and Nurr1 at 0 (sham), 6 h, 12 h, 24 h, 48 h, 72 h, and 7 d in the ipsilateral hemisphere post-HI. The results showed that the expression of MC1R and Nurr1 increased in a time-dependent manner, peaked at 48 h, and then returned to sham levels by 7 d post-HI (*p* < 0.05, Figures 1(a)–1(c)).

### 3.2. Intranasal Administration of BMS-470539 Improved Short-Term Neurological Deficits and Reduced Infarct Area and Body Weight at 48 h Post-HI

**Figure 2:**
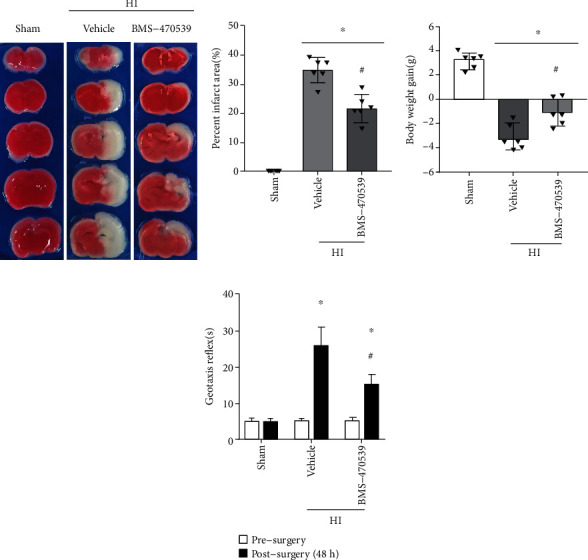
BMS-470539 administration improved short-term neurological deficits and reduced infarct area and body weight loss at 48 h post-HI. (a, b) TTC staining showed that BMS-470539 treatment significantly reduced the percent infarct area when compared with the vehicle. (c) Vehicle-treated pup rats showed to lose significant weight when compared with the sham group and BMS-470539 treatment groups. (d) BMS-470539 treatment significantly improved geotaxis reflex performance when compared with the vehicle-treated pup rats. *n* = 6 per group. Data were represented as mean ± SD. ^∗^*p* < 0.05 versus sham, ^#^*p* < 0.05 versus vehicle. One-way ANOVA, Tukey's post hoc test.

Based on our previous study, the medium (160 *μ*g/kg) dose of BMS-470539 was chosen as the optimal dose, which was used for the short-term outcome [26]. TTC staining results showed that BMS-470539 treatment significantly reduced the percent infarct area at 48 h post-HI compared to the vehicle group (*p* < 0.05, Figures 2(a) and 2(b)). The weight of rat pups was measured, and the rat pups in the vehicle group showed significant body weight loss compared with the sham group and BMS-470539 treatment group at 48 h post-HI (*p* < 0.05, Figure 2(c)). Geotaxis test was used to evaluate the short-term neurological function. In geotaxis test, rat pups performed significantly worse in the vehicle group and BMS-470539 treatment group at 48 h post-HI when compared to the sham group (*p* < 0.05, Figure 2(d)). Intranasal administration of BMS-470539 significantly improved short-term neurological deficits when compared with the vehicle group (*p* < 0.05, Figure 2(d)).

### 3.3. Immunofluorescence Staining Showed MC1R and Nurr1 Colocalization with Neurons at 48 h Post-HI

**Figure 3:**
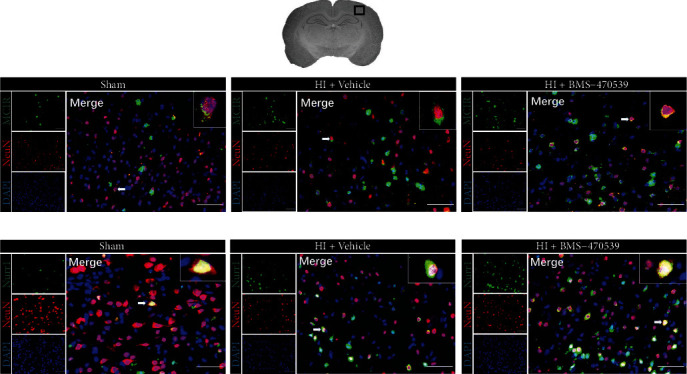
Immunofluorescence staining showed MC1R and Nurr1 colocalization with neurons in the ipsilateral brain hemisphere at 48 h post-HI. Immunofluorescence staining showed that MC1R (a) and Nurr1 (b) expressions on neurons were seen to be higher in the vehicle-treated pup rats compared to the sham group, and a higher expression of MC1R (a) and Nurr1 (b) on neurons after BMS-470359 treatment compared with vehicle. Merge showed the colocalization of MC1R and Nurr1 on neurons. *n* = 2 per group. Neurons were stained red. MC1R and Nurr1 were stained green. DAPI was stained blue. Scale bar = 100 *μ*m.

Immunofluorescence staining showed that MC1R and Nurr1 colocalized with neurons in the sham, vehicle, and treatment groups, and the expression of MC1R and Nurr1 on neurons at 48 h post-HI were significantly increased when compared with the sham group (Figure 3). Furthermore, there was a higher expression of MC1R and Nurr1 on neurons in the BMS-470359 treatment group when compared with the vehicle group (Figure 3).

### 3.4. Intranasal Administration of BMS-470539 Attenuated Neuronal Apoptosis at 48 h Post-HI

**Figure 4:**
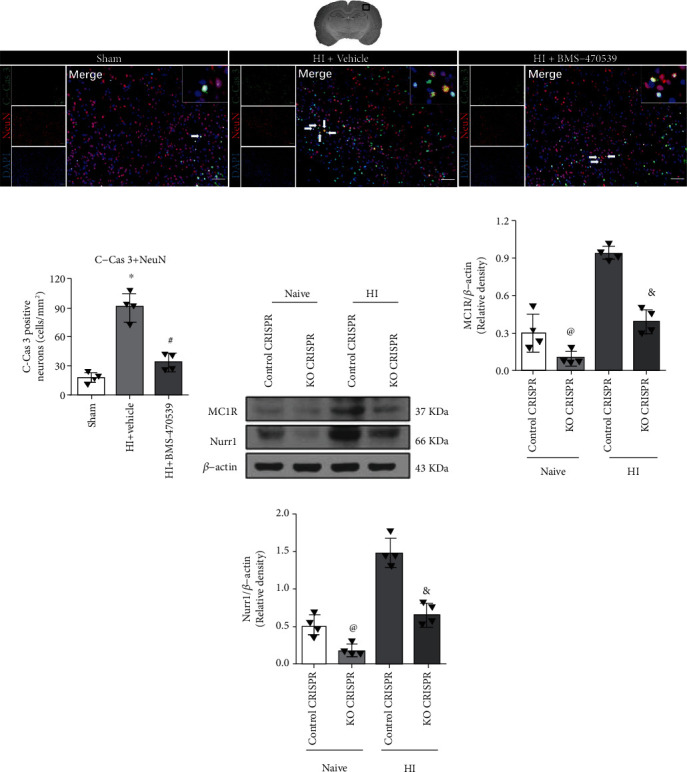
Effects of BMS-470539 treatment on neuronal apoptosis at 48 h post-HI, and the knockout efficiency of MC1R or Nurr1 knockout CRISPR in naive and HI rats. (a, b) Representative microphotographs and quantitative analysis of C-Cas 3-positive neurons in the ipsilateral hemisphere at 48 h post-HI. The number of C-Cas 3-positive neurons significantly increased in the vehicle group compared with the sham group and BMS-470539 treatment group. *n* = 4 per group. C-Cas 3 was green. Neurons were red. Blue was for DAPI. Scale bar = 100 *μ*m. (c, d) Representative Western blot bands and quantitative analysis of MC1R and Nurr1 protein levels in the ipsilateral hemisphere at 48 h post-HI. The expression of MC1R or Nurr1 was significantly reduced by MC1R or Nurr1 knockout CRISPR in naive and HI rats. *n* = 4 per group (data were represented as mean ± SD; ^∗^*p* < 0.05 versus sham; ^#^*p* <0.05 versus HI+vehicle; ^@^*p* < 0.05 versus naive+control CRISPR, ^&^*p* < 0.05 versus HI+control CRISPR; one-way ANOVA, Tukey's post hoc test).

The immunofluorescence staining of C-Cas 3 on neurons was conducted to assess neuronal apoptosis in the ipsilateral hemisphere at 48 h post-HI. Immunofluorescence staining showed the number of C-Cas 3-positive neurons in the vehicle group at 48 h post-HI was significantly increased when compared to the sham group and BMS-470539 treatment group (*p* < 0.05, Figures 4(a) and 4(b)).

### 3.5. In Vivo Knockout of MC1R or Nurr1 Decreased the Expression of MC1R or Nurr1 in Naive and HI Rats

To assess the knockout efficiency of MC1R or Nurr1 KO CRISPR, Western blot was conducted in naive and HI rats. Rat pups were injected with MC1R KO CRISPR, Nurr1 KO CRISPR, or control CRISPR at 48 h before HI induction. Knockout of MC1R or Nurr1 with CRISPR significantly decreased the expressions of MC1R or Nurr1 in naive+KO CRISPR group compared to naive+control CRISPR (*p* < 0.05, Figures 4(c)–4(e)). Similarly, the expressions of MC1R or Nurr1 were significantly decreased in the HI+KO CRISPR group compared to the HI+control CRISPR (*p* < 0.05, Figures 4(c)–4(e)).

### 3.6. Intranasal Administration of BMS-470539 Attenuated Oxidative Stress and Neuronal Apoptosis via MC1R/cAMP/PKA/Nurr1 Signaling Pathway at 48 h post-HI

**Figure 5:**
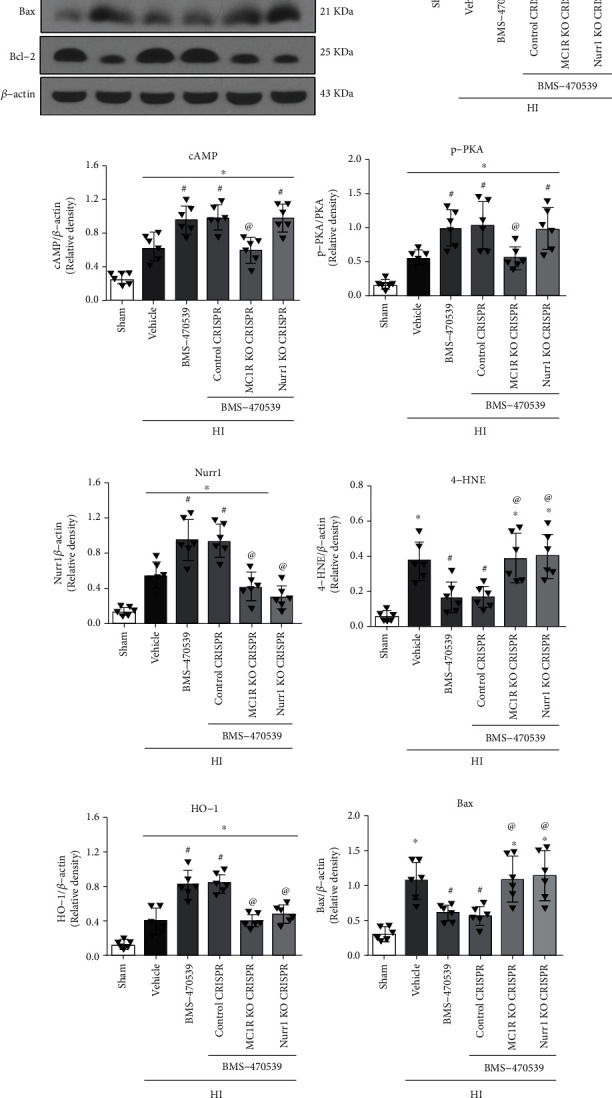
BMS-470539 treatment exerted its antioxidative stress and antiapoptosis effects via MC1R/cAMP/PKA/Nurr1 signaling pathway at 48 h post-HI. (a) Representative Western blot bands. (b–i) Quantification of MC1R, cAMP, p-PKA, Nurr1, 4-HNE, HO-1, Bax, and Bcl-2 expression levels in the ipsilateral hemisphere at 48 h post-HI. BMS-470539 treatment significantly increased the protein levels of MC1R, cAMP, p-PKA, Nurr1, HO-1, and Bcl-2 but significantly decreased the expression of 4-HNE and Bax compared to the HI+vehicle group. Knockout of MC1R or Nurr1 with CRISPR interventions significantly abolished such effects of BMS-470539. *n* = 6 per group. Data was represented as mean ± SD. ^∗^*p* < 0.05 versus sham, ^#^*p* < 0.05 versus HI+vehicle, ^@^*p* < 0.05 HI+BMS-470539 or HI+BMS-470539+control CRISPR; one-way ANOVA, Tukey's post hoc test.

Western blot was performed to investigate the mechanism by which BMS-470539 attenuated oxidative stress and neuronal apoptosis at 48 h post-HI. The Western blot data showed that the expression of MC1R, cAMP, p-PKA, Nurr1, 4-HNE (a marker of oxidative stress), HO-1 (a marker of antioxidative stress response), and proapoptotic protein Bax were significantly increased. In contrast, the antiapoptotic protein, Bcl-2, was significantly decreased in the HI+vehicle group compared with the sham group at 48 h post-HI (*p* < 0.05, Figures 5(a)–5(i)). BMS-470539 treatment resulted in a higher expression of Bcl-2 when compared to the HI+vehicle group at 48 h post-HI (*p* < 0.05, Figures 5(a) and 5(i)). Moreover, BMS-470539 treatment further increased the expression levels of MC1R, cAMP, p-PKA, Nurr1, and HO-1. However, the expression of 4-HNE and Bax were significantly decreased in the HI+BMS-470539 group compared with the HI+vehicle group (*p* < 0.05, Figures 5(a)–5(h)). The expression of MC1R was significantly decreased by knockout of MC1R with CRISPR compared with the HI+BMS-470539 group or HI+BMS-470539+control CRISPR group (*p* < 0.05, Figures 5(a) and 5(b)). Additionally, knockout of MC1R with CRISPR reversed the effects of BMS-470539 on the downstream protein expression. The expressions of cAMP, p-PKA, Nurr1, HO-1, and Bcl-2 were significantly decreased, but the protein levels of 4-HNE and Bax were significantly increased in the HI+BMS-470539+MC1R KO CRISPR group compared with the HI+BMS-470539 group or HI+BMS-470539+control CRISPR group (*p* < 0.05, Figures 5(a) and 5(c)–5(i)).

Consistently, knockout of Nurr1 with CRISPR pretreatment significantly suppressed the expression of Nurr1, HO-1, and Bcl-2 but increased the protein levels of 4-HNE and Bax at 48 h post-HI in the HI+BMS-470539+Nurr1 KO CRISPR group compared with the HI+BMS-470539 group or HI+BMS-470539+control CRISPR group. However, Nurr1 CRISPR did not change the expression of cAMP nor p-PKA (*p* < 0.05, Figures 5(a) and 5(c)–5(i)).

### 3.7. Intranasal Administration of BMS-470539 Reduced Neuronal Degeneration and Suppressed Oxidative Stress at 48 h Post-HI, which Were Abolished by the Knockout of MC1R or Nurr1

**Figure 6:**
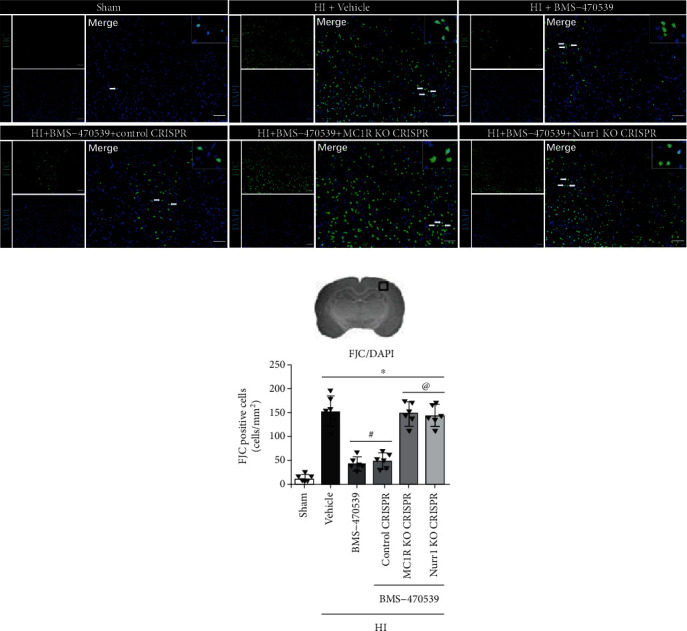
BMS-470539 administration reduced neuronal degeneration at 48 h post-HI, which was reversed by MC1R or Nurr1 knockout CRISPR. (a, b) Representative microphotographs and quantitative analysis of FJC-positive neurons in the ipsilateral hemisphere at 48 h post-HI. *n* = 6 per group. FJC was green. Blue was for DAPI. Scale bar = 100 *μ*m. Data was represented as mean ± SD. ^∗^*p* < 0.05 versus sham; ^#^*p* < 0.05 versus HI+vehicle; ^@^*p* < 0.05 HI+BMS-470539 or HI+BMS-470539+control CRISPR; one-way ANOVA, Tukey's post hoc test.

**Figure 7:**
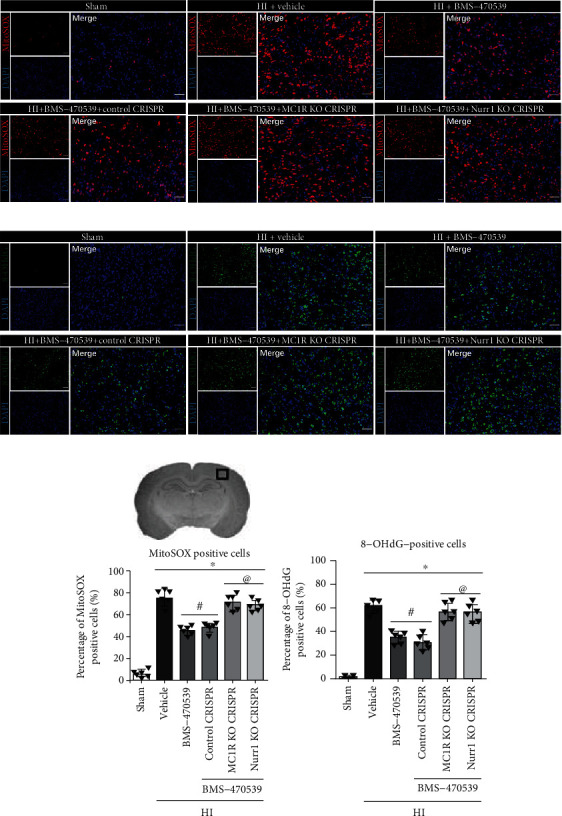
BMS-470539 administration suppressed oxidative stress at 48 h post-HI, which was abolished by MC1R or Nurr1 knockout CRISPR. (a, b) Representative microphotographs of MitoSox (red) and 8-OHdG (green) staining in the ipsilateral hemisphere at 48 h post-HI. Blue was for DAPI. (c, d) Quantitative analysis of MitoSox- and 8-OHdG-positive cells. *n* = 6 per group. Scale bar = 100 *μ*m. Data was represented as mean ± SD. ^∗^*p* < 0.05 versus sham; ^#^*p* < 0.05 versus HI+vehicle; ^@^*p* < 0.05 HI+BMS-470539 or HI+BMS-470539+control CRISPR; one-way ANOVA, Tukey's post hoc test.

FJC (a marker of degenerating neurons), MitoSox (a marker of mitochondrial superoxide), and 8-OHdG (a marker of oxidative stress to DNA) staining were performed to evaluate neuronal degeneration and oxidative stress in the ipsilateral hemisphere at 48 h post-HI. Compared to the sham group, the number of FJC-positive neurons, as well as the percentage of MitoSox- and 8-OHdG-positive cells were significantly increased in the vehicle group at 48 h post-HI (*p* < 0.05, Figures 6(a), 6(b), and 7(a)–7(d)). BMS-470539 treatment significantly reduced the number of FJC-positive neurons and suppressed the expression of MitoSox and 8-OHdG compared with the vehicle group, while knockout of MC1R or Nurr1 with CRISPR interventions significantly abolished the protective effects of BMS-470539 (*p* < 0.05, Figures 6(a), 6(b), and 7(a)–7(d)).

## 4. Discussion

In the present study, we investigated the effects of MC1R activation with BMS-470539 on oxidative stress and neuronal apoptosis in a neonatal HIE rat model and explored the potential underlying mechanisms. We found that the expression of endogenous MC1R and Nurr1 increased in a time-dependent manner post-HI and peaked at 48 h post-HI. MC1R and Nurr1 were colocalized with neurons at 48 h post-HI. In addition, activation of MC1R with BMS-470539 significantly improved short-term neurological deficits, reduced infarct area, and attenuated neuronal apoptosis by decreasing the number of C-Cas 3-positive neurons at 48 h post-HI. Mechanistically, BMS-470539 treatment upregulated the protein levels of MC1R, cAMP, p-PKA, Nurr1, HO-1, and Bcl-2 but downregulated the protein levels of 4-HNE and Bax, as well as reduced FJC-positive cells, MitoSox-positive cells, and 8-OHdG-positive cells in the ipsilateral hemisphere at 48 h post-HI. The knockout of MC1R or Nurr1 by specific MC1R CRISPR and Nurr1 CRISPR interventions abolished the beneficial effects of BMS-470539 on neuronal degeneration, oxidative stress, and neuronal apoptosis. These observations indicated that BMS-470539 might attenuate oxidative stress and neuronal apoptosis at 48 h post-HI, which was, at least in part, mediated by upregulating the MC1R/cAMP/PKA/Nurr1 signaling pathway.

Mounting evidence has suggested that oxidative stress exacerbates neuronal apoptosis and degradation, which is an underlying mechanism leading to brain dysfunction and subsequent neurological dysfunction post-HI [9–12]. MC1R belongs to the melanocortin receptor subtype family. It has been shown to exert a neuroprotective effect by inhibiting oxidative stress and neuronal apoptosis in central nervous system diseases, including SAH, ICH, and traumatic brain injury [21, 22, 24]. In the present study, the expression of endogenous MC1R significantly increased over time and peaked at 48 h post-HI. Our previous study demonstrated that the upregulation of endogenous MC1R as a protective factor post-HI may indicate its protective response to harmful stimuli in the acute stage post-HI [26]. In addition, we found that MC1R was expressed on neurons at 48 h post-HI, consistent with previous studies [21, 24].

Nurr1, a transcription factor, provides a neuroprotective effect by inhibiting the transcription of genes coding for inflammation, apoptosis, oxidative stress, and mitochondrial dysfunction, as well as stimulating the transcription and expression of neurotrophic factors [51–53]. Previous studies found that Nurr1 exerted neuroprotective, anti-inflammatory, antioxidative stress, and antiapoptotic effects in vivo and in vitro Parkinson's disease models [29, 51]. Our previous study demonstrated that Nurr1 was expressed on microglia and attenuated neuroinflammation in a rat model of HIE [26]. It has also been reported that activation of Nurr1 with amodiaquine attenuated neuroinflammation and neuronal apoptosis after SAH in rats [54]. Recently, increased expression of Nurr1 reportedly protected dopaminergic neurons by regulating mitochondria-mediated apoptotic molecules, such as Bcl-2, Bax, Cyt-c, cleaved caspase-9, and cleaved caspase-3 [52]. Microarray analysis revealed that overexpression of Nurr1 confers resistance to oxidative stress by downregulating the expression of cleaved caspase-3 and other apoptotic factors in neural stem cells [28]. Moreover, Nurr1 has also been reported to have effects against apoptosis in other tissues [30, 31, 55–58]. It has been proven that knockdown of Nurr1 promoted apoptosis in various types of cancer cells, including pancreatic ductal adenocarcinoma cells, colorectal carcinoma cells, bladder cancer cells, and cervical cancer cells [55–57]. Activating Nurr1 also inhibited apoptosis and amends redox balance via GDNF/AKT pathway in a model of hepatic ischemia/reperfusion [58]. Together, the reported scientific literature suggested that Nurr1 is a promising therapeutic target against oxidative stress and apoptosis. In the present study, the expression levels of endogenous Nurr1 increased significantly along with MC1R and peaked at 48 h post-HI. The increased expression of MC1R and Nurr1 may suggest the activation of protective factors after an injury as the body attempts to maintain homeostasis. However, the extent of these increases was not sufficient to offset the damage caused by harmful factors. We also observed that Nurr1 was expressed on neurons at 48 h post-HI, which is consistent with previous reports [54, 59].

BMS-470539, a novel-selective agonist of MC1R, exerts anti-inflammatory, antioxidative, and antiapoptotic effects in lung inflammation, human chondrocyte cell line, SAH, and HIE [20, 24–26, 60]. We previously demonstrated the anti-inflammatory and neuroprotective effects of BMS-470539 after neonatal HIE in rats [26]. However, MC1R activation with BMS-470539 on oxidative stress and neuronal apoptosis in neonatal HIE have not been explored. In the present study, BMS-470539 treatment significantly attenuated oxidative stress and neuronal apoptosis, as seen by an increase in HO-1 and Bcl-2 protein levels and a decrease in the levels of 4-HNE and Bax, as well as MitoSox-positive cells, 8-OHdG-positive cells, FJC-positive cells, and C-Cas 3-positive neurons at 48 h post-HI.

In our previous study, we used three doses, low (50 *μ*g/kg), medium (160 *μ*g/kg), and high (500 *μ*g/kg), to evaluate the optimal dose of BMS-470539 [26]. Based on the infarct area, weight loss, and short-term neurological function evaluation at 48 h post-HI, the medium dose (160 *μ*g/kg) of BMS-470539 was chosen as the optimal dose for long-term outcome and mechanism studies, as previously reported [26]. We previously found that BMS-470539 treatment significantly improved the long-term neurological performance on the foot-fault, rotarod, and Morris water maze test at 28 days post-HI [26]. Therefore, we also chose the optimal dose (160 *μ*g/kg) of BMS-470539 for the short-term outcome and mechanism studies in the present study. Specifically in the present study, we found that BMS-470539 treatment improved short-term neurological deficits, reduced infarct area and body weight loss at 48 h post-HI. Similarly, previous studies suggested that BMS-470539 improved short- and long-term neurological deficits after SAH [20, 24]. Based on the above results, we speculated that BMS-470539 treatment improved neurological deficits and reduced infarct area post-HI, which was, at least in part, mediated by inhibiting oxidative stress and neuronal apoptosis.

We further explored the possible molecular mechanism underlying the antioxidative stress and antiapoptotic effects of activation of MC1R with BMS-470539. After binding to melanocortin, MC1R upregulates cAMP expression by activating adenylate cyclase [37, 61, 62]. The MC1R-mediated cAMP signaling has been identified as a major mechanism in regulating pigmentation, adaptive tanning, and melanoma resistance [61, 63, 64]. A major transduction pathway for cAMP signaling is via the cAMP-dependent PKA signaling pathway [65, 66]. It has been reported that cAMP binds and activates PKA via the cAMP/PKA signaling to increase the level of PKA phosphorylation [67, 68]. Coincidentally, PKA was proposed as an upstream regulatory molecule of Nurr1, upregulating the expression of Nurr1 through a PKA-dependent manner in vivo and in vitro experiments [33, 69, 70]. In the present study, we found that BMS-470539 treatment significantly upregulated the protein levels of MC1R, cAMP, p-PKA, and Nurr1 in the ipsilateral hemisphere at 48 h post-HI. The knockout of MC1R with specific MC1R CRISPR reversed the antioxidative stress and antiapoptotic effects of BMS-470539 by decreasing MC1R expression level and its downstream molecules of cAMP, p-PKA, Nurr1, HO-1, and Bcl-2, while increasing the expressions of 4-HNE, Bax, MitoSox-positive cells, 8-OHdG-positive cells, and FJC-positive cells at 48 h post-HI. Moreover, although the knockout of Nurr1 with specific Nurr1 CRISPR had no effects on the expression of cAMP and PKA's phosphorylation, it also significantly abolished antioxidative stress and antiapoptotic effects of BMS-470539. Collectively, BMS-470539 attenuates oxidative stress and neuronal apoptosis, at least in part, via upregulation of the MC1R/cAMP/PKA/Nurr1 signaling pathway post-HI.

There are several limitations to this study. First, previous studies and our data showed that the MC1R was expressed on neurons [21, 24]. Future study is needed to investigate the effect of MC1R activation with BMS-470539 on neuronal pyroptosis. Second, previous studies have demonstrated that MC1R activation may exert antioxidative stress and antiapoptotic effects through other signaling pathways, such as AMPK/SIRT1/PGC-1*α* pathway and PI3K/Akt/Nrf2 pathway [21, 24]. In the present study, we only studied the cAMP/PKA/Nurr1 signaling pathway. Future studies are needed to explored other pathways after HIE. Third, little is known regarding the downstream pathways of Nurr1-mediated antioxidative stress and antiapoptosis. Therefore, future studies should focus on the underlying mechanisms of how Nurr1 inhibited oxidative stress and apoptosis.

## 5. Conclusion

Our study showed that BMS-470539 administration attenuated oxidative stress and neuronal apoptosis and improved neurological deficits, at least in part, by activating the MC1R/cAMP/PKA/Nurr1 signaling pathway in a neonatal HI rat model. Therefore, early administration of BMS-470539 may be a promising therapeutic and preventive strategy for infants with HIE.

## Data Availability

The data used to support the findings of this study are available from the corresponding author on reasonable request.
